# Thermoelectric Transport in Nanocomposites

**DOI:** 10.3390/ma10040418

**Published:** 2017-04-15

**Authors:** Bin Liu, Jizhu Hu, Jun Zhou, Ronggui Yang

**Affiliations:** 1 Center for Phononics and Thermal Energy Science, School of Physics Science and Engineering, Tongji University, Shanghai 200092, China; 10liubin@tongji.edu.cn (B.L.); hujizhu0318@tongji.edu.cn (J.H.); 2 Department of Mechanical Engineering, University of Colorado, Boulder, CO 80309, USA; ronggui.yang@colorado.edu

**Keywords:** nanocomposites, thermoelectric materials, transport

## Abstract

Thermoelectric materials which can convert energies directly between heat and electricity are used for solid state cooling and power generation. There is a big challenge to improve the efficiency of energy conversion which can be characterized by the figure of merit (*ZT*). In the past two decades, the introduction of nanostructures into bulk materials was believed to possibly enhance *ZT*. Nanocomposites is one kind of nanostructured material system which includes nanoconstituents in a matrix material or is a mixture of different nanoconstituents. Recently, nanocomposites have been theoretically proposed and experimentally synthesized to be high efficiency thermoelectric materials by reducing the lattice thermal conductivity due to phonon-interface scattering and enhancing the electronic performance due to manipulation of electron scattering and band structures. In this review, we summarize the latest progress in both theoretical and experimental works in the field of nanocomposite thermoelectric materials. In particular, we present various models of both phonon transport and electron transport in various nanocomposites established in the last few years. The phonon-interface scattering, low-energy electrical carrier filtering effect, and miniband formation, etc., in nanocomposites are discussed.

## 1. Introduction to Thermoelectricity

Thermoelectricity, which directly converts thermal energy and electricity, is expected to play an increasing role in meeting the world’s rapid growing energy demand and in the thermal management [1,2,3,4,5,6,7,8,9,10]. The efficiency of energy conversion in thermoelectric (TE) materials is described by the dimensionless figure of merit (*ZT*)

(1) $$ZT=\frac{S^{2}\sigma T}{\kappa_{c}+\kappa_{p}},$$

where *S*, *σ*, *T*, $\kappa_{c}$, and $\kappa_{p}$ are, respectively, the Seebeck coefficient, electrical conductivity, absolute temperature, electronic thermal conductivity due to the contribution of electrical carriers, and lattice thermal conductivity due to the contribution of phonons. $S^{2}\sigma$ in the numerator is usually mentioned as the power factor [11,12,13]. The efficiency of the TE device is increased by making *ZT* as large as possible since the efficiency of power generation ($\chi_{power}$) and the coefficient of performance (*COP*) of refrigeration device are

(2) $$\chi_{power}=\chi_{c}\frac{\sqrt{1+Z\bar{T}}-1}{\sqrt{1+Z\bar{T}}+T_{c}/T_{H}},$$

(3) $$COP=\frac{T_{c}\left(\sqrt{1+Z\bar{T}}-T_{c}/T_{H}\right)}{(T_{H}-T_{c})\left(\sqrt{1+Z\bar{T}}+1\right)},$$

where $\chi_{c}=\left(T_{H}-T_{c}\right)/T_{H}$ is the Carnot efficiency, $\bar{T}=\left(T_{c}+T_{H}\right)/2$ is the mean temperature, $T_{H}$ denotes the temperature of the hot end, and $T_{c}$ denotes the temperature of the cold end. It is straightforward that a high *ZT* material requires both large power factor and low total thermal conductivity ($\kappa_{c}+\kappa_{p}$). In conventional bulk TE materials, the interrelationship between *S*, *σ*, and $\kappa_{c}$ results in a difficulty of the independent control of these variables. Therefore, *ZT* of the best bulk TE material was hardly able to exceed one for 40 years since 1950s. The state-of-the-art bulk materials at different temperature regimes are as follows, *ZT* ≈ 1 at low temperature regime (200 K–400 K) in alloys of Bi_2_Te_3_ and Sb_2_Te_3_ for use in refrigeration [14], *ZT* ≈ 1.2 in *p*-type alloy (GeTe)_0.85_(AgSbTe)_0.15_ and *ZT* ≈ 1.8 in *p*-type PbTe-based alloy for use in mid-temperature (400 K–800 K) power generation [15,16], *ZT* ≈ 1 in *n*-type Si_0.8_Ge_0.2_ alloy for use in high-temperature (above 800 K) power generation [17]. Various classes of materials are also widely studied: magnesium and manganese group IV compounds such as Mg_2_Si , Mg_2_Ge and MnSi*_x_* [18,19,20], rock-salt structural materials such as SnTe and SnSe [21,22], tetrahedrites such as Cu_12−*x*_M*_x_*(Sb,As)_4_S_13_ [23], half-Heusler compounds such as ZrNiSn-based alloy [24], Skutterudite-based materials such as CoSb_3_ and CeFe_4_Sb_12_ [25], clathrate such as (Ba,Sr)_8_(Al,Ga)_16_(Si,Ge,Sn)_30_ [26], CsBi_4_Te_6_ [27], Tl_9_BiTe_6_ [28], In_4_Se_3−δ_ [29], and Cu_2−*x*_Se [30,31], and so on.

From the 1990s, the introduction of nanostructures has shown a promising way to independently control *S*, *σ*, and $\kappa_{c}$ [1] through quantum confinement effect, and to reduce the lattice thermal conductivity $\kappa_{p}$ due to the phonon-interface scattering [1,32,33,34] for the next generation of high efficiency TE materials. For example, *ZT* ≈ 2.4 at room temperature has been reported by Venkatasubramanian et al. [35] in *p*-type Bi_2_Te_3_/Sb_2_Te_3_ thin film TE materials. *ZT* ≈ 1.6 at room temperature has also been reported by Harman et al. [36] in PbSeTe-based quantum dot (QD) superlattices materials. Since the Seebeck coefficient *S* represents the average entropy of electrical carriers [37], band engineering by properly manipulating the density of states (DOS) of carriers (electrons, holes, and small polarons, et al., depends on materials) that is responsible for electrical current could significantly enhance the Seebeck coefficient. It is straightforward to utilize the low-dimensional structures such as superlattices [35,38], nanowires [39,40], and quantum wells [41,42] to control DOS. DOS distortions induced by introducing resonant energy levels through doping [43] and convergence of many valleys by tuning the doping and composition [16] in bulk materials are newly proposed ways to engineer the DOS for high TE efficiency. It is also proposed that low-energy carriers can be filtered through incoherent or random interfaces [44,45,46] which presents another way to enhance *S* significantly. 

## 2. Nanocomposites for Thermoelectricity

Nanocomposite (NC) is one candidate that could be easily synthesized to possibly achieve all three effects mentioned above: reduction of lattice thermal conductivity, engineering DOS, and low-energy carrier filtering [47,48,49]. Over the past few years, lots of works have been carried out to improve the TE efficiency using various NC materials. A review of the studies to control and understand the formation and the transport properties of the NC materials is given by Medlin and Snyder [50]. The TE properties of NC materials based on different traditional TE materials such as Bi_2_Te_3_, PbTe, and SiGe are reported experimentally. We review the experimental achievements of NC materials in detail in the following subsections.

### 2.1. Bismuth Telluride Based Nanocomposites

Bi_2_Te_3_-based materials are most widely used as commercial TE materials for solid-state refrigeration and thermal management near room temperature [51,52,53]. The maximum *ZTs* of *p*-type (Bi*_x_*Sb_1−*x*_)_2_Te_3_ alloy and *n*-type Bi_2_(Te*_y_*Se_1−*y*_)_3_ alloy are about 1 for a long time. It was recently reported that the peak *ZT* of Bi_2_Te_3_-based NCs has been enhanced to 1.3–1.8 [54,55,56,57,58].

**Figure 1:**
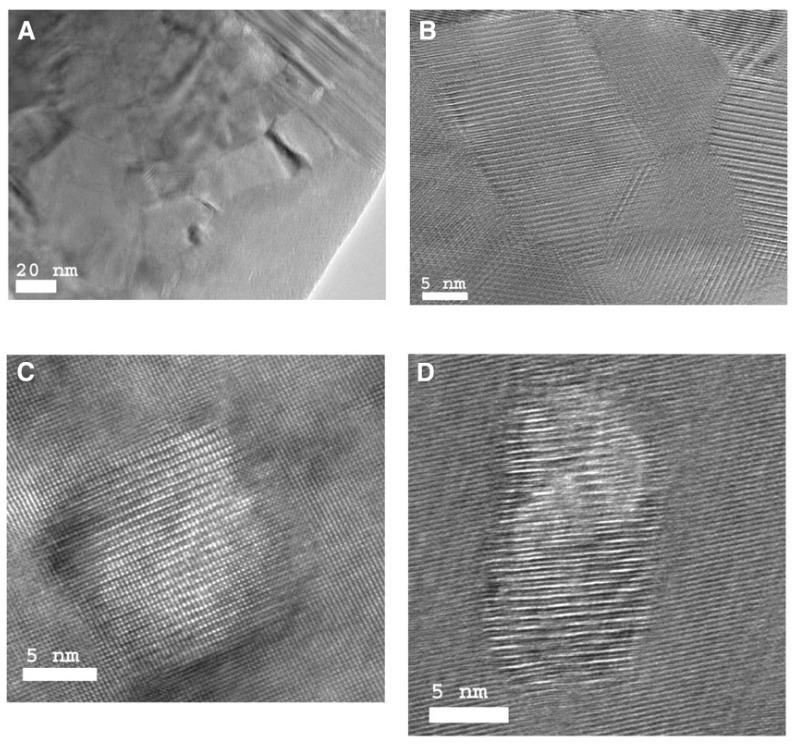
Transmission electron microscope (TEM) images showing the microstructures of hot-pressed Bi*_x_*Sb_2−*x*_Te_3_-based nanocomposite (NC) materials. (**A**) Low-magnification image showing the nanograins. (**B**) High-magnification image showing the grain boundaries. High-magnification images showing the nanodots in matrix (**C**) without boundaries and (**D**) with small angle grain boundaries. Reprinted with permission from Reference [54]. Copyright 2008, American Association for the Advancement of Science.

**Figure 2:**
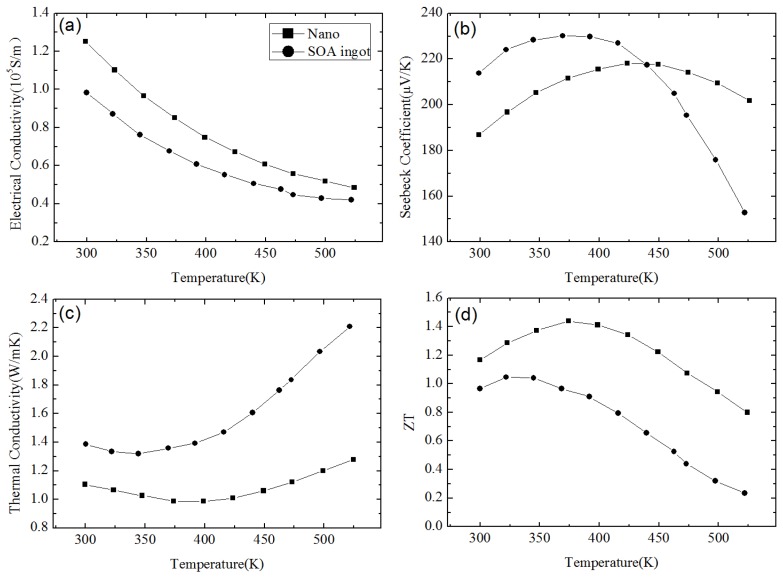
Temperature dependence of (**a**) electrical conductivity; (**b**) Seebeck coefficient; (**c**) thermal conductivity; and (**d**) *ZT* of *p*-type Bi_0.5_Sb_1.5_Te_3_-based NC material in comparison with that of a state-of-the-art (SOA) ingot. Reprinted with permission from Reference [54]. Copyright 2008, American Association for the Advancement of Science.

**Figure 3:**
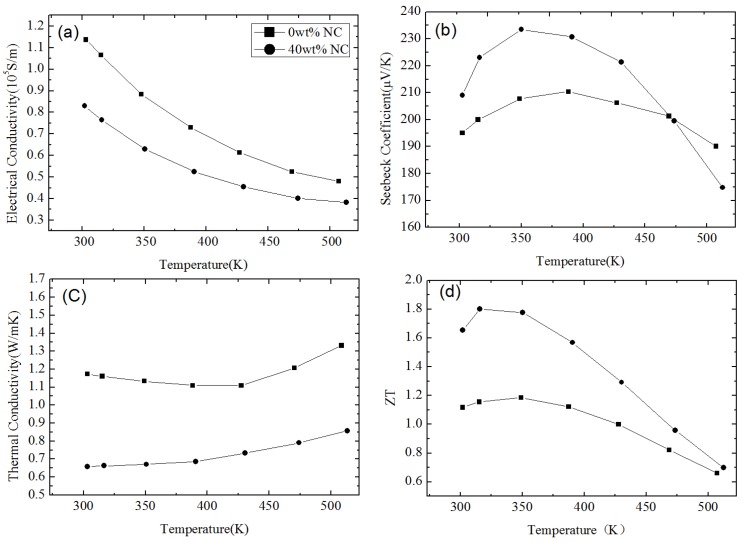
Temperature dependence of (**a**) electrical conductivity; (**b**) Seebeck coefficient; (**c**) thermal conductivity; and (**d**) *ZT* of p-type Bi_0.4_Sb_1.6_Te_3_-based NC material with 40 wt% of nanoinclusions in comparison with that of NC material with 0 wt% of nanoinclusions. Reprinted with permission from Reference [63]. Copyright 2010, American Institute of Physics.

Figure 1 shows the nanostructures of a *p*-type Bi_0.5_Sb_1.5_Te_3_ NCs sample synthesized by Poudel et al. [54] by using the ball-milling and hot-pressing method. Nanoinclusions with random shape can be seen in the figure. The measured size of the nanoinclusions in this NCs material is between 5 nm to 50 nm while the average value is about 20 nm. In spite of the reduction of carrier mobility due to additional carrier-interface scattering, an increase of electrical conductivity is found, as shown in Figure 2a because of the increase of hole concentration partly due to the formation of nanoprecipitates in NCs. Figure 2b shows the slight decrease of Seebeck coefficient and the peak value shifts from 370 K to a higher temperature 450 K. The thermal conductivity of NCs is reduced a lot at all temperatures as shown in Figure 2c because of the abundant interface between nanoinclusions and matrix in NCs. Therefore, an enhancement of *ZT* is obtained in Figure 2d due to the reduction of thermal conductivity without significantly changing the electronic transport properties. *ZT* ≈ 1.2 at room temperature is obtained and the peak *ZT* appears to be 1.4 near 373 K. Following the first work done by Poudel et al. [54], Cao et al. [56] found that *p*-type (Bi*_x_*Sb_1−*x*_)_2_Te_3_-based NCs with laminated nanostructures prepared by hydrothermal synthesis and hot-pressing method exhibit a maximum *ZT* ≈ 1.47 at 480 K. Xie et al. reported that *ZT* ≈ 1.56 at 300 K could be obtained in *p*-type Bi_0.52_Sb_1.48_Te_3_-based NCs [59] and *ZT* ≈ 1.5 at 390 K could be obtained in *p*-type Bi_0.48_Sb_1.52_Te_3_-based NCs [60] which were fabricated by a melt spinning technique followed by a quick spark plasma sintering procedure. Dou et al. fabricated Bi_0.4_Sb_1.6_Te_3_-based NCs embedded with amorphous SiO_2_ nanoparticles and found that the enhancement of *ZT* was attributed to the increase in Seebeck coefficient and reduction in thermal conductivity [61]. Guo et al. [62] fabricated Bi_0.4_Sb_1.6_Te_3_-based NCs incorporated with small proportion (0.3 vol%) of nanophase Cu_2_Se and found that *ZT* ≈ 1.6 could be obtained at 488 K. Fan et al. [63] reported the TE transport in a *p*-type Bi_0.4_Sb_1.6_Te_3_-based NCs fabricated by a rapid and high throughput method of mixing nanoparticles obtained though melt spinning as shown in Figure 3. The electrical conductivity of 40 wt% NC in Figure 3a is reduced that is different from that in Figure 2a. The Seebeck coefficient in Figure 3b is slightly enhanced. The thermal conductivity in Figure 3c which is below 0.8 W/(m·K) is even smaller than the minimum of thermal conductivity in Figure 2c. The extremely low thermal conductivity results in *ZT* = 1.8 at 316 K as shown in Figure 3d. Furthermore, (Bi*_x_*Sb_1−*x*_)_2_Te_3_ NC materials doped with small amount of PbTe were studied by Ebling et al. [64], the presence of PbTe is helpful to enhance *ZT*.

**Figure 4:**
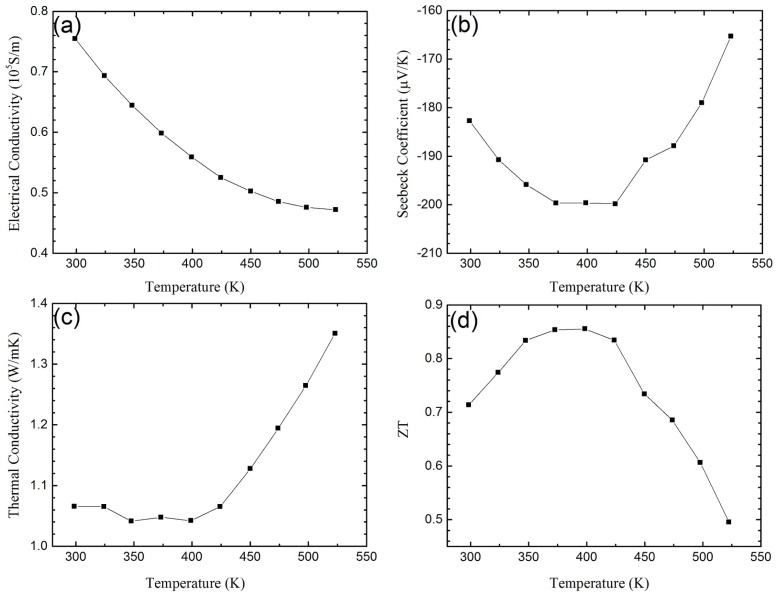
Temperature dependence of (**a**) electrical conductivity; (**b**) Seebeck coefficient; (**c**) thermal conductivity; and (**d**) *ZT* of *n*-type Bi_2_Te_3−*x*_Se*_x_*-based NC material. Reprinted with permission from Reference [65]. Copyright 2010, American Chemical Society.

*n*-Type Bi_2_Te_3−*x*_Se*_x_* NC materials can also be fabricated with similar techniques such as ball-milling and hot pressing [65,66,67,68,69,70]. Figure 4 shows the TE properties of *n*-type Bi_2_Te_3−*x*_Se*_x_* NC prepared in the same way as *p*-type NCs [65]. The electrical conductivity shown in Figure 4a is low while the Seebeck coefficient shown in Figure 4b is maintained in comparison with the bulk material. The low thermal conductivity shown in Figure 4c leads to a peak of *ZT* slightly below 1. Very recently, Soni et al. [71,72] reported that the TE properties could be enhanced in Bi_2_Te_3−*x*_Se*_x_* nanoplatelet-based NCs. Besides that, Xiong et al. reported that at 370 K *ZT* ≈ 1.59 could be obtained in *p*-type Bi_0.5_Sb_1.5_Te_3_-based NCs and *ZT* ≈ 1 could be obtained in *n*-type Bi_2_Te_2.7_Se_0.3_-based NCs which were fabricated by introducing the liquid-phase-sintering process to the bottom-up approach [73].

### 2.2. Lead Telluride Based Nanocomposites

**Figure 5:**
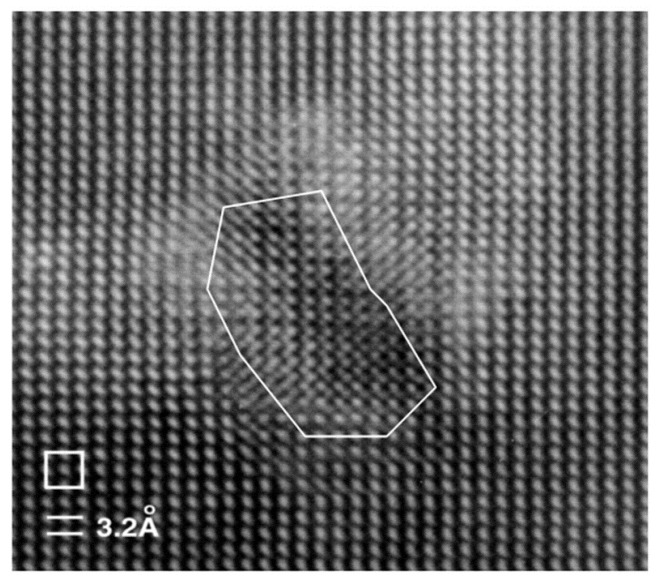
TEM image of AgPb_18_SbTe_20_ NC material showing an Ag-Sb-rich nanodot. Reprinted with permission from Reference [74]. Copyright 2004, American Association for the Advancement of Science.

**Figure 6:**
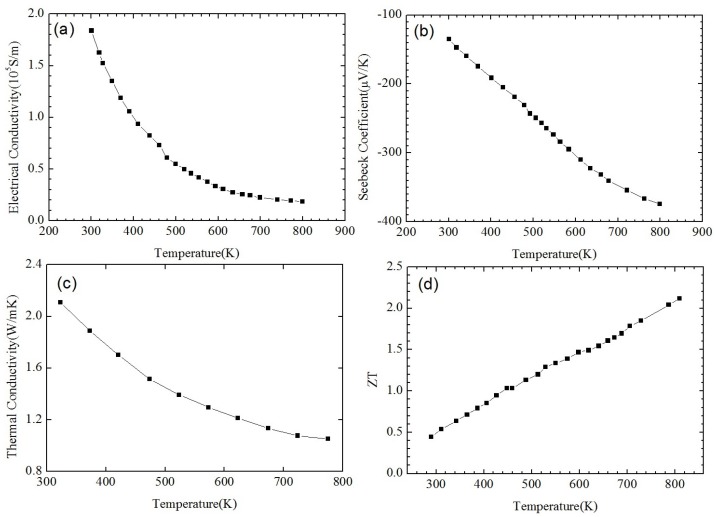
Temperature dependence of (**a**) electrical conductivity; (**b**) Seebeck coefficient; (**c**) thermal conductivity; and (**d**) *ZT* of AgPb_18_PbTe_20_-based NC material. Reprinted with permission from Reference [74]. Copyright 2004, American Association for the Advancement of Science.

PbTe is one of the best TE materials used in mid-temperature regime. PbTe-AgSbTe_2_ (or AgPb_m_SbTe_2+m_)-based NC materials were first studied by Hsu et al. [74]. They found a maximum *ZT* ≈ 2.2 at *T* = 800 K in AgPb_m_SbTe_2+m_ with nanoscale region of the crystal structure that was Ag-Sb-rich in composition [75,76]. Figure 5 shows the TEM image of AgPb_18_SbTe_20_-based NCs with Ag-Sb-rich nanometer sized nanodots. Figure 6 shows the TE transport properties in these NCs. Extremely large power factor 28 μW/cm·K^2^ at 700 K is obtained due to the electrical conductivity shown in Figure 6a which is about 0.25 × 10^5^ S/m and large Seebeck coefficient shown in Figure 6b which is −335 μV/K. Together with low thermal conductivity as shown in Figure 6c, *ZT*~2.2 at 800 K is obtained as shown in Figure 6d. Wang et al. [77] also found a maximum power factor of 17.66 μW/cm·K^2^ at 673 K in Ag_0.8_Pb_22_SbTe_20_ NCs which corresponds to a high *ZT* = 1.37. Paul et al. [78] reported a power factor 18.78 μW/cm·K^2^ at 500 K in NCs with Ag rich nanodots embedded in PbTe matrix.

**Figure 7:**
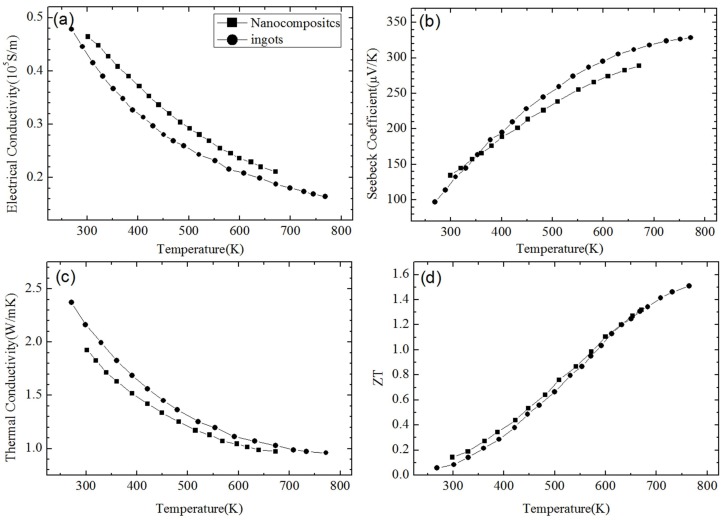
Temperature dependence of (**a**) electrical conductivity; (**b**) Seebeck coefficient; (**c**) thermal conductivity; and (**d**) *ZT* of *p*-type PbTe-based NC material in comparison with that of ingot [48,79]. Reprinted with permission from Reference [48]. Copyright 2010, WILEY-VCH Verlag GmbH & Co.

Besides PbTe-AgSbTe_2_-based NC materials, PbTe-based NC materials are also fabricated. Figure 7 shows the TE transport properties of *p*-type PbTe NCs with Tl doping compared with the PbTe ingots reported by Yu et al. [48,79]. The electrical conductivity in NCs is increased at all temperatures as shown in Figure 7a and the Seebeck coefficient is reduced when the temperature is over 350 K as shown in Figure 7b. Then, an increase of power factor can be found at low temperature and a decrease of power factor can be found at high temperature. The thermal conductivity in NCs is smaller than that in ingots as shown in Figure 7c. Therefore, *ZT* in Figure 7d is enhanced at low temperature and changes slightly at high temperature. Moreover, Kim et al synthesized the PbTe-based heterogeneous nanocomposites by mixing the nanodot nanocomposite and the nanograined nanocomposites and reported that *ZT* ≈ 2.0 could be obtained at 773 K [80]. Martin et al. [81] prepared *p*-type PbTe NCs from nanocrystals synthesized using an aqueous solution-phase reaction. Heremans et al. [82] fabricated the *p*-type PbTe based NCs with both nanosized grains and EuTe nanoinclusions. They studied the low-energy carrier filtering effect which induced the enhancement of Seebeck coefficient. They also fabricated the PbTe NCs containing nanometer-sized metal Pb precipitates [83]. Vinies et al. [84] reported a comparison between the measured and calculated electrical transport properties for both *n*-type and *p*-type PbTe/PbSe nanodot superlattices. A reduction of carrier mobility is observed while the Seebeck coefficient is unaffected.

### 2.3. Silicon Germanium Based Nanocomposites

SiGe alloys are the major TE materials for power generation at high temperature. It has been used in radio-isotope thermoelectric generators (RTGs) for deep-space explorations. The peak *ZT* reaches 1 in *n*-type SiGe bulk alloy [85] and 0.65 in *p*-type SiGe bulk alloy [17].

**Figure 8:**
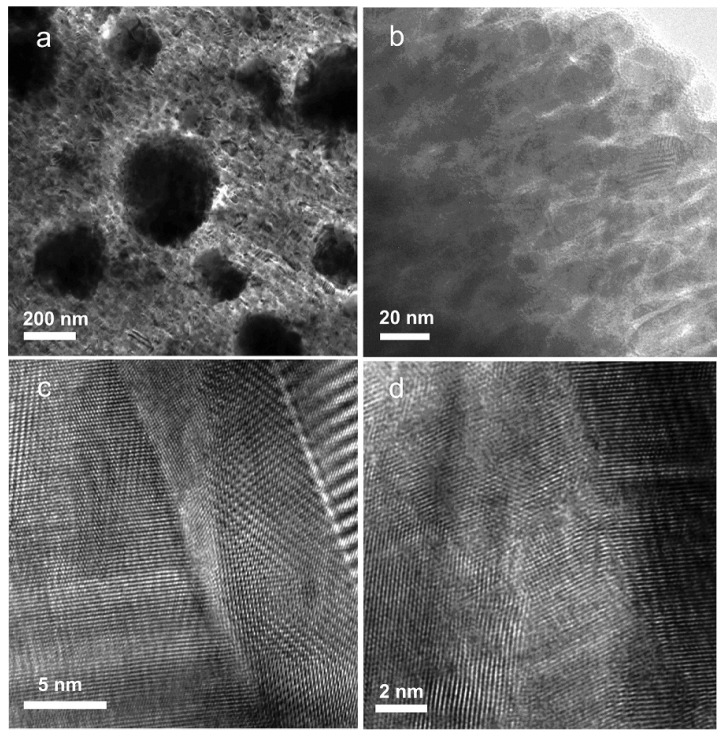
TEM images of SiGe NC material under (**a**) low-magnification, (**b**) medium-magnification, (**c**,**d**) high-magnification. Reprinted with permission from Reference [86]. Copyright 2008, American Chemical Society.

**Figure 9:**
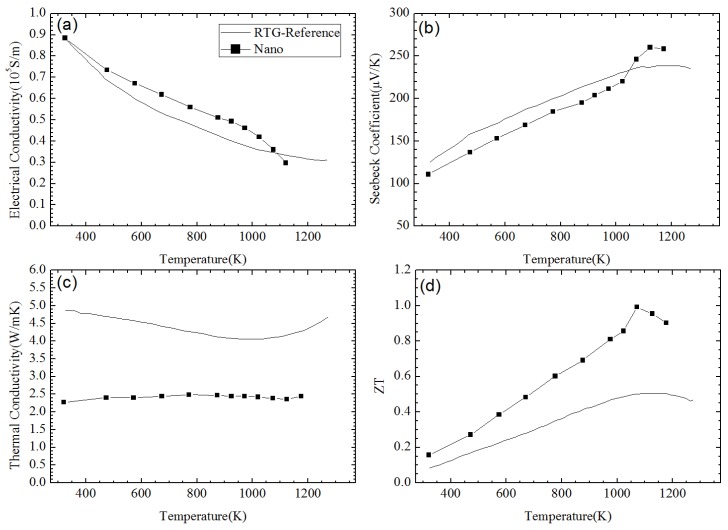
Temperature dependence of (**a**) electrical conductivity; (**b**) Seebeck coefficient; (**c**) thermal conductivity; and (**d**) *ZT* of *p*-type Si_80_Ge_20_-based NC material in comparison with the *p*-type SiGe bulk alloy used in radio-isotope thermoelectric generators (RTGs) [45]. Reprinted with permission from Reference [86]. Copyright 2008, American Chemical Society.

**Figure 10:**
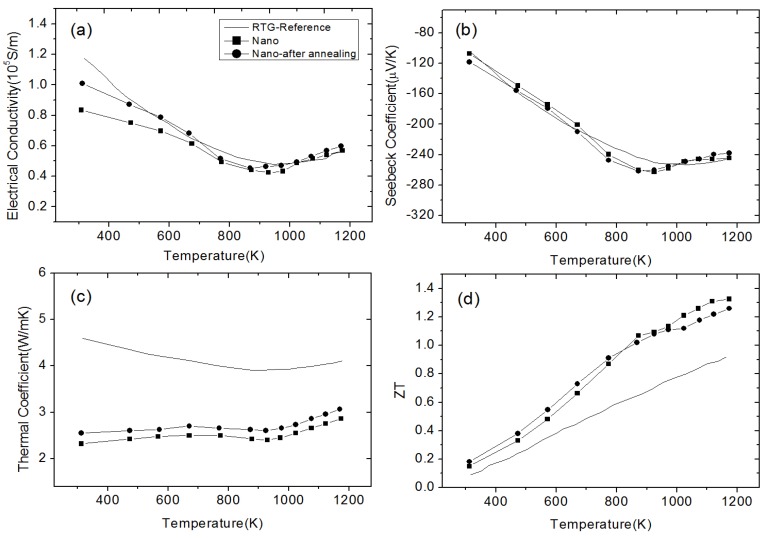
Temperature dependence of (**a**) electrical conductivity; (**b**) Seebeck coefficient; (**c**) thermal conductivity; and (**d**) *ZT* of *n*-type Si_80_Ge_20_-based NC material and the sample after annealing in comparison with the *n*-type SiGe bulk alloy used in RTGs. Reprinted with permission from Reference [87]. Copyright 2008, American Institute of Physics.

SiGe alloy-based NCs material has been fabricated using ball-milling and hot pressing method by Joshi et al. [86]. Figure 8 shows the TEM images of the nanostructures of the *p*-type SiGe hot pressed NCs in Reference [86]. The TE transport properties of Si_80_Ge_20_ NCs are plotted in Figure 9 in comparison with the *p*-type SiGe bulk alloy used in RTGs. Figure 9a,b show that the electrical conductivity and the Seebeck coefficient in NC samples can be either larger or smaller than that in RTGs materials at different temperature. The power factor could be comparable to that in RTGs. Due to the significant reduction of thermal conductivity as shown in Figure 9c, peak *ZT* about 0.95 can be obtained in Figure 9d. Wang et al. [87] reported the enhanced TE performance in *n*-type SiGe NCs with and without annealing in comparison with the *n*-type SiGe bulk alloy used in RTGs as shown in Figure 10. Similar to *p*-type NCs, the electrical conductivity shown in Figure 10a and the Seebeck coefficient shown in Figure 10b slightly change in NCs. The thermal conductivity as shown in Figure 10c decreases from about 4.5 W/(m·K) to about 2.5 W/(m·K) at all temperatures. Then *ZT* in NCs reaches 1.3 around 1200 K which is much higher than 1 in *n*-type SiGe bulk alloy used in RTGs as shown in Figure 10d.

Moreover, SiGe NCs with other nanoinclusions are also studied. Yu et al. [88] demonstrated that modulation-doping could increase *ZT* in Si_86.25_Ge_13.75_P_1.05_ NCs through increasing the carrier mobility as well as power factor rather than reducing thermal conductivity. Zhu et al. [89] reported the increased phonon-interface scattering when low concentration of Ge was doped in nanostructured Si could enhance *ZT*. Nozariasbmarz et al. presented that the incorporation of metallic iron silicide in SiGe NCs could effectively improve the electrical conductivity and thus increase *ZT* over the temperature range of 800–950 °C [90]. Zamanipour and Vashaee [91] investigated the Si_0.8_Ge_0.2_ based NCs embedded with CrSi_2_ nanoinclusions from both experimental and theoretical approaches. They found that the power factor was enhanced due to the enhancement in charge carrier mobility.

### 2.4. Other Nanocomposites

**Figure 11:**
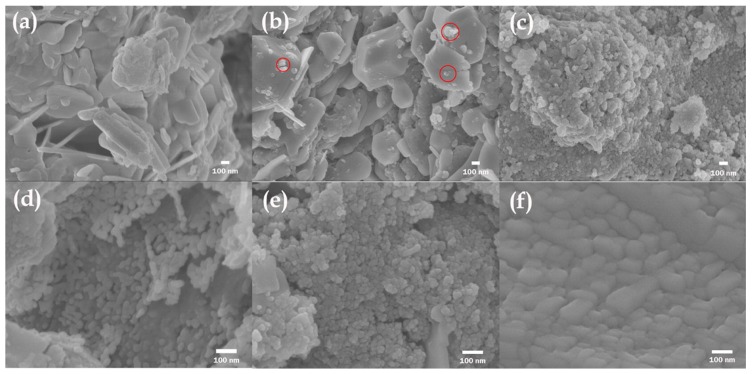
Scanning electron microscopy (SEM) images of (**a**) ZnO; (**b**) Zn_0.99_Al_0.01_O; (**c**) Zn_0.98_Al_0.02_O; (**d**) Zn_0.98_Al_0.02_O/rGO (1.5 wt%); (**e**) Zn_0.98_Al_0.02_O/rGO (2.5 wt%); and (**f**) Zn_0.98_Al_0.02_O/rGO (3.5 wt%). Red circles indicate the Al in AZO as confirmed by energy-dispersive X-ray (EDX) measurement. Reprinted with permission from Reference [99]. Copyright 2015, American Chemical Society.

Recently, ZnO has become a potential candidate for high temperature TE materials but suffers from low electrical conductivity [92]. Many ZnO-based NCs such as Al-doped ZnO (AZO), Ga-doped ZnO (GZO), and Al and Ga co-doped ZnO have been investigated for increasing the electrical conductivity and thus realization of high temperature TE power generation, in which *ZT* are obtain to be 0.3–0.65 at 1000 K [93,94,95,96,97,98]. Liang et al. [99] proposed that the AZO-based NCs embedded with reduced graphene oxides (rGOs) could effectively modulate the carrier concentration and thus optimize the electrical conductivity. The morphologies of the pure ZnO, AZO alloys, and Zn_0.98_Al_0.02_O/rGO hybrids is shown in Figure 11, as measured by field-effect scanning electron microscopy (SEM). Simultaneously, the increased interface between the AZO and rGOs could remarkably reduce the lattice thermal conductivity due to the increasing phonon-boundary scattering [99]. Other materials such as manganese silicide based NCs [100] and magnesium silicide based NCs [101,102,103], polycrystalline SnSe embedded with PbTe nanoinclusions [104], Skutterudites CoSb_3_ compounds [105] and Yb*_x_*Co_4_Sb_12_ alloy [106] based NCs, *n*-type half-Heuslers alloy Hf_0.75_Zr_0.25_NiSn_0.99_Sb_0.01_ based NCs [107], *p*-type half-Heuslers alloy Zr_0.5_Hf_0.5_CoSb_0.8_Sn_0.2_ based NCs [108] and Zr_0.25_Hf_0.25_NiSn based NCs [109], BiCuSeO-based NCs [110] and InGaAs alloy embedded with ErAs nanoparticles [111,112] are also studied. Furthermore, some non-TE materials like carbon nanotube [113], C_60_ [114,115], graphene [116,117], SiC [118] and platinum nanocrystals [119] are used as nanoconstituents to embed into Bi_2_Te_3_ and Sb_2_Te_3_ based materials to enhance *ZT*.

Organic NCs could be used to enhance the TE efficiency [120]. They are possibly advantageous compared with the inorganic materials due to the fact that they are much easier and cheaper to synthesize. Tunable physical and chemical properties in a fairly large range through simple modifications of their molecular structures provide great material flexibility to meet the requirements of the potential applications. For TE properties in organic materials, large electrical conductivity and large Seebeck coefficient have been reported in conducting polymers when the materials are appropriately doped [121,122]. Combined with the low thermal conductivity, large *ZT* values can be speculated. For example, a *ZT* value of ~0.38 has been reported for iodine-doped polyacytelene [123] and the value is 0.25 in poly(3,4-ethylenedioxythiophene) (PEDOT) [124]. The TE properties in nanostructured organic materials such as organic films [125], chain-like polymer structures [126], organic hybrid materials [127], and self-assembled molecular nanowires that are composed of one-dimensional (1D) stacks of planar building blocks loosely held together [128] have been studied experimentally. There are not many theoretical works for potentially high-efficiency organic TE materials. Casian [129] predicted that very large *ZT* at room temperature was possible for quasi-1D organic crystals by using the Boltzmann transport equations of conducting electrons. Wang et al. [130] used the first-principles calculations coupled with the Boltzmann transport theory to study the TE properties in pentacene and rubrene crystals and *ZT* ranging from 0.8 to 1.1. Wang et al. [131] applied the Holstein small polaron model to study the TE properties in the quasi-1D molecular nanowires. The study of TE properties in organic NCs is still in the infant stage, lots of efforts are needed in this field.

**Figure 12:**
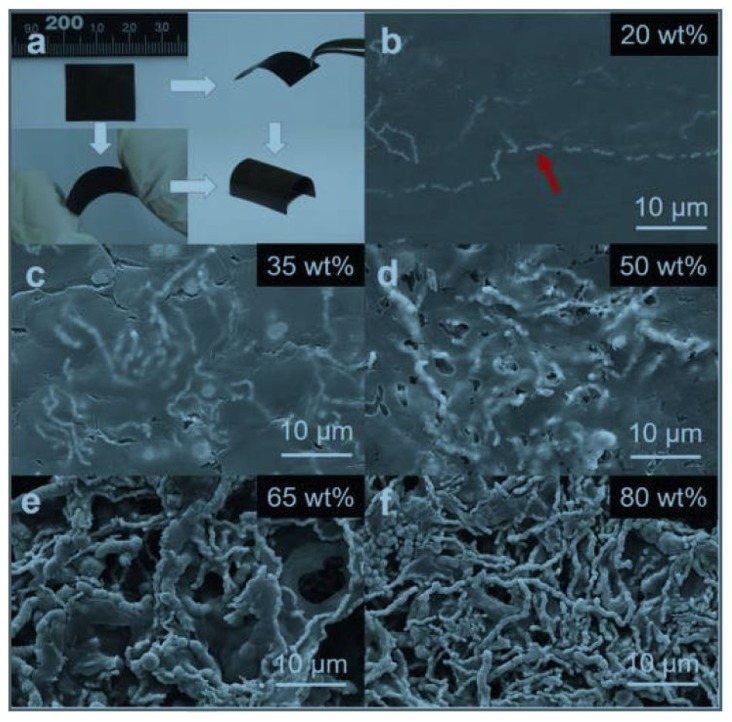
(**a**) Typical photographs of highly bendable Ni/ polymer polyvinylidene fluoride (PVDF) thermoelectric (TE) NC film. (**b**–**f**) Top-view SEM images of the Ni/PVDF TE NCs with different contents of Ni nanowires. Ni nanowires are denoted by the red arrow. Reprinted with permission from Reference [146]. Copyright 2017, WILEY-VCH Verlag GmbH & Co KGaA, Weinheim.

Recently, organic/inorganic NCs provide an opportunity for enhancing TE performance by combining the advantages of low thermal conductivity and high power factor from the organic and inorganic components, respectively. Some inorganic materials such as carbon nanotubes [132,133], traditional inorganic TE fibers [134,135], metallic nanowires [136,137,138], and so on, have been applied as inorganic components to achieve high enhancement of the Seebeck coefficient and/or electrical conductivity in the conductive polymers. Meanwhile, many conductive inorganic/insulating organic NCs have been studied [139,140,141], in which the infinite conductive path is formed when the volumetric fraction of the inorganic materials is beyond the critical volume fraction according to the percolation theory [142,143,144]. The electrons can easily pass through the conductive path, and thus, the universal scaling law is obeyed for the electrical conductivity but not for the thermal conductivity, since the thermal energy flows in both organic and inorganic materials [145]. Furthermore, the newly raised research work has demonstrated that the combination of Ni nanowires and insulating polymer polyvinylidene fluoride (PVDF) could exhibit outstanding *n*-type TE properties [146]. Figure 12a reveals that the as-fabricated Ni/PVDF TE NC films are highly bendable and hard to deform. The Top-view SEM images of the Ni/PVDF TE NCs with different contents of Ni nanowires are shown in Figure 12b–f. The incorporation of Ni nanowires provides a high power factor of 200 μW/m·K^2^.

## 3. Modeling of Phonon Transport

In this section, we review the transport properties of phonons in NCs that are studied by a wide variety of models. In NCs, it has been pointed out that the Fourier’s law breaks down in predicting the lattice thermal conductivity [147]. The calculation of lattice thermal conductivity of NCs beyond the Fourier’s theory was carried out through solving the phonon Boltzmann transport equation (BTE) and the phonon Monte Carlo in a series of papers by Yang et al. [148,149,150,151,152]. However, the BTE calculation is difficult to implement in all but some particular materials with simple geometries [153,154] and the computational cost of the Monte Carlo simulation is quite high. Satyala and Vashaee [155,156] used the Steigmer and Abeles model based on the Callaway method [157] to model phonon transport with the BTE and relaxation time approximation. The phonon-phonon, phonon-electron, point defect, and grain boundary scattering mechanisms are incorporated into their model. A similar model was also developed by Mingo et al. [158] to calculate the thermal conductivity in the SiGe alloy containing Si nanoparticles.

**Figure 13:**
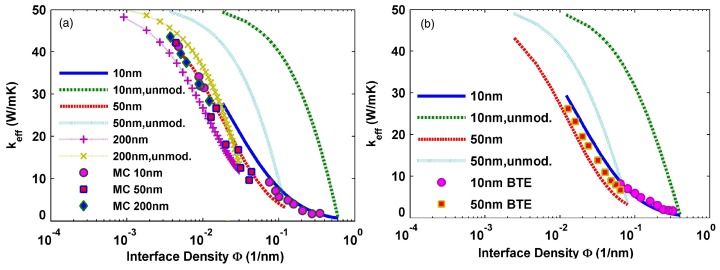
Thermal conductivity as a function of interface density for a SiGe NC with spherical inclusions predicted by the modified and unmodified effective medium approximation (EMA) formulae in comparison with the data from (**a**) Monte Carlo simulation and (**b**) Boltzmann equation. Reprinted with permission from Reference [159]. Copyright 2007, American Institute of Physics.

The modified effective medium approximation (EMA) method which gives a closed-form expression for the thermal conductivity in NCs with spherical nanoparticles is developed by Minnich and Chen [159]. This method makes the calculation much simpler. Such a modified method is based on the EMA method that is used to calculate the thermal conductivity of NCs, which considers the thermal boundary resistance [160]. The effective thermal conductivity of NCs as a function of interface density Φ and nanoparticle diameter a can be written as follows:

(4) $$\kappa_{eff}\left(\Phi ,a\right)=\frac{1}{3}c_{h}\nu_{p,h}\frac{1}{\left(1/\Lambda_{h}\right)+\left(\Phi /4\right)}\times \frac{\kappa_{p}\left(a\right)\left(1+2\varphi \left(\Phi ,a\right)\right)+2\kappa_{h}\left(\Phi \right)+2\left(\Phi a/6\right)\left[\kappa_{p}\left(a\right)\left(1-\varphi \left(\Phi ,a\right)\right)-\kappa_{h}\left(\Phi \right)\right]}{\kappa_{p}\left(a\right)\left(1+2\varphi \left(\Phi ,a\right)\right)+2\kappa_{h}\left(\Phi \right)-\left(\Phi a/6\right)\left[\kappa_{p}\left(a\right)\left(1-\varphi \left(\Phi ,a\right)\right)-\kappa_{h}\left(\Phi \right)\right]}.$$

where $\Lambda_{h}$, $c_{h}$, and $\nu_{p,h}$ are the phonon mean free path, specific heat, and phonon group velocity in host material, respectively.$\;\Phi =4\pi {\left(a/2\right)}^{2}/D_{0}^{3}$, $\varphi \left(\Phi ,a\right)=\kappa_{h}\left(\Phi \right)/\left(a/2\right)$, *D_0_* is the effective cell length, $\kappa_{p}\left(a\right)$ is the particle thermal conductivity, and $\kappa_{h}\left(\Phi \right)$ is the host material thermal conductivity. Figure 13 shows the effective thermal conductivity as a function of interface density for different sizes of nanoparticles in SiGe NCs with Si nanoparticles embedded in Ge host calculated using modified EMA and unmodified EMA in comparison with Monte Carlo simulation and BTE. The results calculated from modified EMA are in good agreement with results from Monte Carlo simulations and the solutions of BTE.

Ordonez et al. [161] extended the modified EMA method to consider the NCs with spheroidal nanoparticles whose size and shape could affect the thermal conductivity. Poon and Limtragool [162] extended the modified EMA method to investigate concentrated NCs in which the inter-particle phonon scattering processes beyond the independent nanoparticles phonon scattering should be considered. They found that the thermal conductivity varies more rapidly with the volumetric fraction of nanoinclusions in comparison with original modified EMA method.

## 4. Modeling of Electrical Carrier Transport

**Figure 14:**
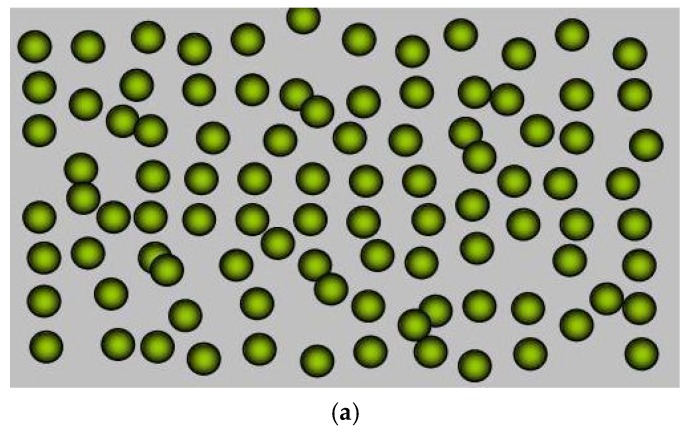

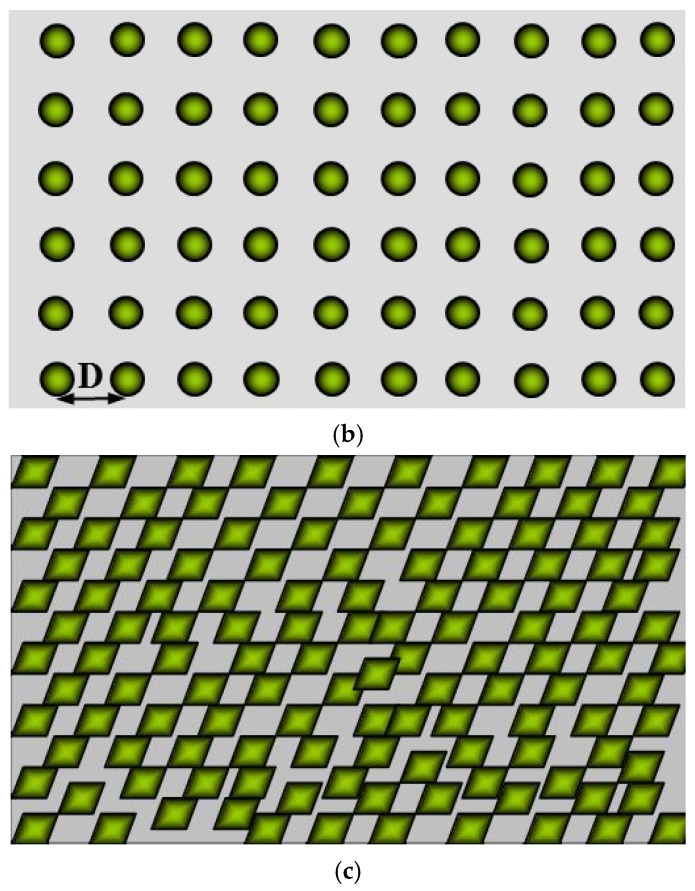
(**a**) Nanoconstituents (nanoparticles or nanowires) randomly embedded in a matrix (host) material, noted as random particle-host type NCs; (**b**) Nanoconstituents periodically embedded in a matrix material, noted as ordered particle-host type NCs. In these two types of NCs, any two points in the matrix can be connected without encountering any nanoconstituents; (**c**) Mixture of different kinds of nanoconstituents, noted as particle-particle-type NCs. In this type of NCs, two arbitrary points in two separated nanostructures of the same material cannot be connected without encountering the other kind of nanoconstituent. Reprinted with permission from Reference [163]. Copyright 2010, American Physical Society.

In this section, we review the model for electrical carrier transport in NCs. First, NCs are classified for convenience in Figure 14. Figure 14a shows the NCs with nanoconstituents (nanoparticles or nanowires) randomly embedded in a matrix (host) material that is called “*random particle-host-type*” NCs. Nanoconstituents periodically embedded in a matrix material as shown in Figure 14b is called “*ordered particle-host-type*” NCs. In these two types of NCs, any two points in the matrix can be connected without encountering any nanoconstituents, i.e., there is a bypass for electron transport to go around the nanoconstituents. Another type of NCs is a mixture of different kinds of nanoconstituents as shown in Figure 14c which is called “*particle-particle-type*” NCs. In this type of NCs, two arbitrary points in two separated nanostructures of the same material cannot be connected without encountering the other kind of nanoconstituents. Different theories should be used to describe different types of NCs.

### 4.1. Boltzmann Transport Equations for Electrical Transport

For electron transport, a transport theory is proposed based on BTE in NCs beyond the conventional bulk TE transport model which was proposed by Bergman et al. [164,165] in the 1990s using effective transport matrix and field decoupling transformation method. The electrical transport theory which is under the framework of BTE and relaxation time approximation (RTA) usually starts with Kane model [166] in which multiple bands are considered. The dispersion relation of each carrier pocket in each band can be written as:

(5) $$\frac{\hbar^{2}k_{i,j,∥}^{2}}{2m_{i,j,∥}^{*}}+\frac{\hbar^{2}k_{i,j,⊥}^{2}}{2m_{i,j,⊥}^{*}}=\gamma \left(E_{i,j}\right)=E_{i,j}\left(1+E_{i,j}/E_{g}\right).$$

Here i=(1,2,⋯) represents the band index, j=(e,h) represents the electrons (*e*) and holes (*h*), $m_{i,j,∥}^{*}\left(m_{i,j,⊥}^{*}\right)$ is the effective mass in a carrier pocket in parallel (perpendicular) direction near the band edge and $k_{i,j,∥}$($k_{i,j,⊥}$) is the corresponding wave vector. It is noted that $m_{i,j}^{*}={\left(m_{i,j,∥}^{*2}m_{i,j,⊥}^{*}\right)}^{1/3}$ is the density-of-states effective mass of each carrier pocket, and $m_{d,i,j}^{*}={\left(N\right)}^{2/3}m_{i,j}^{*}$ is the density-of-states effective mass of each energy band with degeneracy *N*. The non-parabolicity of the band is taken into account through the energy term $\left(E_{i,j}\right)=E_{i,j}\left(1+E_{i,j}/E_{g}\right)$, where $E_{i,j}/E_{g}$ is the non-parabolicity factor and $E_{g}$ is the energy gap.

Then the electrical conductivity σ, Seebeck coefficients *S*, and electronic thermal conductivity $\kappa_{c}$ of both electrons and holes can then be calculated [11]:

(6) $$\sigma_{\xi }=\sum_{j}\sigma_{\xi ,j},\;\sigma_{\xi ,j}=\sum_{i}\frac{q_{j}^{2}}{3\pi^{2}m_{\xi ,i,j}^{*}}{\left(\frac{2k_{B}Tm_{d,i,j}^{*}}{\hbar^{2}}\right)}^{3/2}L_{i,j}^{0};$$

(7) $$S_{\xi }=\sum_{j}\frac{S_{j}\sigma_{\xi ,j}}{\sigma_{\xi ,j}},\;S_{j}=\frac{k_{B}}{q_{j}}\left(\frac{\sum_{i}\frac{{\left(m_{d,i,j}^{*}\right)}^{\frac{3}{2}}}{m_{\xi ,i,j}^{*}}L_{i,j}^{1}}{\sum_{i}\frac{{\left(m_{d,i,j}^{*}\right)}^{\frac{3}{2}}}{m_{\xi ,i,j}^{*}}L_{i,j}^{0}}-\eta_{i,j,F}\right);$$

$$\kappa_{c,\xi }=\frac{\sigma_{\xi ,e}\sigma_{\xi ,h}}{\sigma_{\xi ,e}+\sigma_{\xi ,h}}{\left(S_{e}-S_{h}\right)}^{2}T+\sum_{j}\kappa_{c,\xi ,j},$$

(8) $$\kappa_{c,\xi ,j}=\sum_{i}\frac{k_{B}^{2}T}{3\pi^{2}m_{\xi ,i,j}^{*}}{\left(\frac{2k_{B}Tm_{d,i,j}^{*}}{\hbar^{2}}\right)}^{3/2}\times \left(L_{i,j}^{2}-\frac{\sum_{i^{\prime }}\frac{{\left(m_{d,i^{\prime },j}^{*}\right)}^{3/2}}{m_{\xi ,i,j}^{*}}L_{i^{\prime },j}^{1}}{\sum_{i^{\prime }}\frac{{\left(m_{d,i^{\prime },j}^{*}\right)}^{3/2}}{m_{\xi ,i,j}^{*}}L_{i^{\prime },j}^{0}}L_{i,j}^{1}\right).$$

Here ξ=(∥,⊥), $q_{j}$ denotes the charge of carrier, $\eta_{i,j}=E_{i,j}/k_{B}T$, $\eta_{i,e,F}=\left(\mu -E_{g}-E_{i,e,0}\right)/k_{B}T$, $\eta_{i,h,F}=\left(-\mu -E_{i,h,0}\right)/k_{B}T$, $\eta_{g}=E_{g}/k_{B}T$, and $\gamma \left(\eta_{i,j}\right)=\eta_{i,j}\left(1+\eta_{i,j}/\eta_{g}\right)$, respectively. The integrations in Equations (6)–(8) can be written as

(9) $$L_{i,j}^{v}=\int_{0}^{\infty }\eta_{i,j}^{v}\frac{\gamma^{3/2}\left(\eta_{i,j}\right)}{\gamma^{\prime }\left(\eta_{i,j}\right)}\tau_{i,j}^{tot}\left(-\frac{\partial f}{\partial \eta_{i,j}}\right)d\eta_{i,j},$$

where *f* is the equilibrium Fermi-Dirac distribution. $\tau_{i,j}^{tot}$ is the total relaxation time which is calculated by the Mathiessen’s rule assuming that the scattering events are independent of each other:

(10) $$\frac{1}{\tau_{i,j}^{tot}}=\frac{1}{\tau_{i,j}}+\frac{1}{\tau_{b,i,j}}\;,$$

where $\tau_{i,j}$ is the relaxation time of intrinsic scattering mechanisms in bulk alloys such as electron-phonon scattering and electron-impurity scattering, and $\tau_{b,i,j}$ is the carrier-interface relaxation time which will be discussed in detail in next subsection. The detailed expressions of the electron-phonon scattering relaxation time and carrier-impurity scattering relaxation time are given in Reference [165,167,168].

### 4.2. Relaxation Time Model for Carrier-Interface Scattering

**Figure 15:**
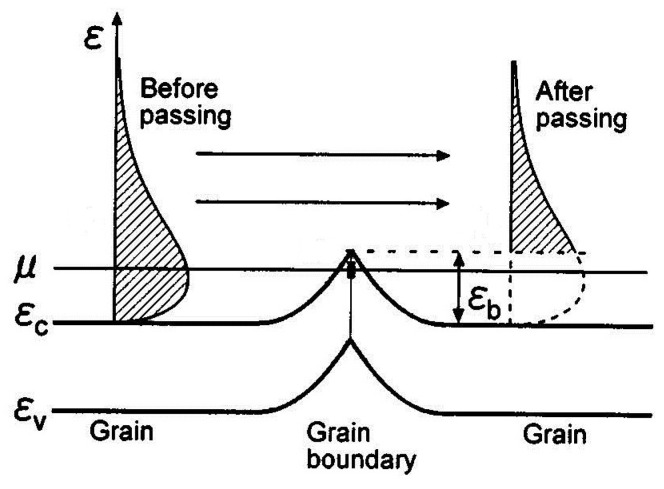
Schematic diagram of potential barrier scattering at grain boundary. A potential barrier of height $\varepsilon_{b}$ is formed at grain boundary where the *μ* is chemical potential, $\varepsilon_{c}$ and $\varepsilon_{\nu }$ are the band edges of conduction band and valence band. The carriers whose energies are lower than the barrier height are filtered while the carriers whose energies are higher than the barrier height can easily pass through the potential barrier. Reprinted with permission from Reference [172]. Copyright 2002, American Institute of Physics.

**Figure 16:**
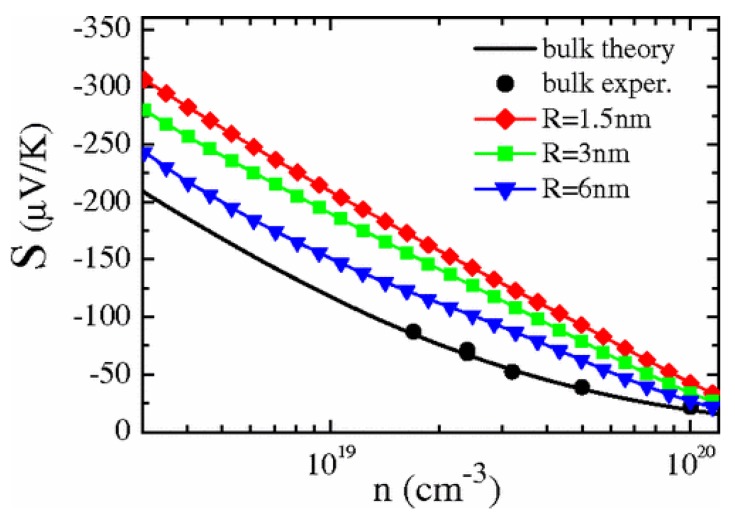
Seebeck coefficient calculated by partial wave method for PbTe NC with metallic nanoinclusions as a function of doping density for different nanoinclusion radius in comparison with the bulk values. Reprinted with permission from Reference [176]. Copyright 2008, American Physical Society.

In NCs, the Seebeck coefficient could be increased since the average entropy (or average energy) carried by each carrier is enhanced by energy-selective filtering effect [44,168,169]. Such an effect filters the electrical carriers whose energy are lower than the energy barrier while the carriers whose energy are higher than the energy barrier $\varepsilon_{b}$ can easily pass through, as shown in Figure 15. A carrier-interface relaxation time is first introduced to describe the interface scattering experienced by the carriers in NCs by assuming a one-dimensional rectangular potential barrier in some models [12,47,170,171,172]. Yang and Chen study the TE transport properties for Si_0.8_Ge_0.2_ alloy-based NCs as a function of potential barrier height [173] through writing the carrier-interface relaxation time in a simple way:

$$\frac{1}{\;\tau_{b}}=\frac{6\sqrt{2E/m^{*}}}{a}\;\mathrm{when}\;E<\varepsilon_{b},$$

(11) $$\frac{1}{\tau_{b}}=0\;\mathrm{when}\;E>\varepsilon_{b},$$

where $\varepsilon_{b}$ is the energy barrier height, and subscript *i*, *j* are dropped for simplicity in the following discussion. Going a step further, Minnich et al. [174] considered the interface scattering using a charge-trapping model. The filtering effect of Shottky barriers potential induced by trapping states in grain boundaries is studied using one-band effective mass model within the Landauer formulism by Bachmann et al. [175]. Faleev et al. [176] and Zebarjadi et al. [177] used the partial wave method and the Born approximation, which was applicable when the concentration of nanoinclusions was very low, to calculate the electronic transport cross section $\sigma_{t}$ and then obtained the carrier-interface relaxation time through the relation:

(12) $$\frac{1}{\tau_{b}}=N_{i}\nu \sigma_{t},$$

where $N_{i}$ is the concentration of nanoinclusions. Figure 16 shows the enhanced Seebeck coefficient calculated in Reference [176] in PbTe NCs with spherical metallic nanoinclusions for different radius and fixed volumetric fraction. Smaller nanoinclusions lead to larger absolute Seebeck coefficient because of the larger interface density. When the concentration of nanoinclusion is high, the nanoinclusions should not be treated independently; an effective medium theory based on the coherent potential approximation similar to that used in disordered system is proposed by Zebarjadi et al. [178,179]. A perturbation theory was also used by Yang and Qin [180] to analytically calculate the carrier-interface scattering probabilities to obtain the relaxation time. All the above carrier-interface scattering models are used for particle-host type NCs. 

**Figure 17:**
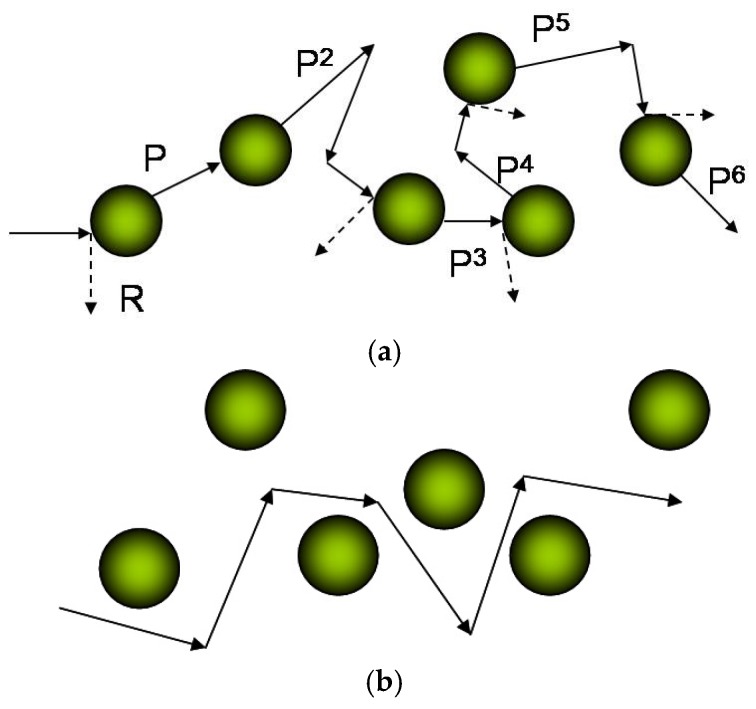
(**a**) Schematic diagram of the random trajectory of a carrier transmit through or scatter by nanoconstituents one by one with transmission probability *P* and reflection probability *R =* 1 − *P*. (**b**) Schematic diagram of open path that the carrier does not meet any nanoconstituents. This open path can exist only in particle-host type. In particle-particle type, there is no such path because of the absence of topological connectedness. Reprinted with permission from Reference [163]. Copyright 2010, American Physical Society.

All the above models are useful in particle-host type NCs. Zhou et al. [163] proposed a carrier-interface scattering model which could be used in both the particle-host type NCs and particle-particle NCs. They considered the relaxation time as the average flying time between two scattering events. The average free path length could be evaluated by averaging on distance ι which is the distance between two successive boundaries and the carrier transmission probability *P* through a potential barrier. When the carriers travel in crystals with random trajectories, they will encounter the interface of nanoinclusion randomly. During its random trajectory, the carrier will transmit through the potential barrier of the nanoinclusions one by one with transmission coefficient *P*, or be scattered away by these barriers with reflection coefficient 1 − *P* as shown in Figure 17a [181]. Particularly, if the carrier does not meet any potential barrier during its random trajectory, we called this trajectory an open path, as shown in Figure 17b. 

Then, in particle-host type NC, the relaxation time due to interface scattering is:

(13) $$\tau_{b}=\frac{\iota_{\alpha }}{\nu_{\alpha }}\frac{1}{1-P}+\frac{\iota_{\beta }}{\nu_{\beta }}\frac{P}{1-P},$$

where $\nu_{\alpha }\left(\nu_{\beta }\right)$ is the velocity of the carrier in matrix (nanoinclusions), $\iota_{\alpha }=a\left(1-x\right)/6x$, $\iota_{\beta }=a/6$ for spherical nanoinclusions, *x* is the volumetric fraction of nanoinclusions. *α* and *β* are the index of different constituents materials in NC.

In particle-particle type NC, because of the absence of connectedness, there is no open path, in other words, the path shown in Figure 17b does not exist. Then the relaxation time due to interface scattering is slightly different from the particle-host type given in Equation (13):

(14) $$\tau_{b}=\frac{\iota_{\alpha }}{\nu_{\alpha }}\frac{P}{1-P}+\frac{\iota_{\beta }}{\nu_{\beta }}\frac{P}{1-P}.$$

The carrier transmission probability *P* through the energy barrier is a function of the carrier energy, the height and geometry of each potential barrier. From the energy dependence of *P*, one can obtain the effective barrier height $\varepsilon_{b}$ near which *P* changes from 0 to 1 dramatically. Detailed calculation of *P* can be found in Reference [163]. 

**Figure 18:**
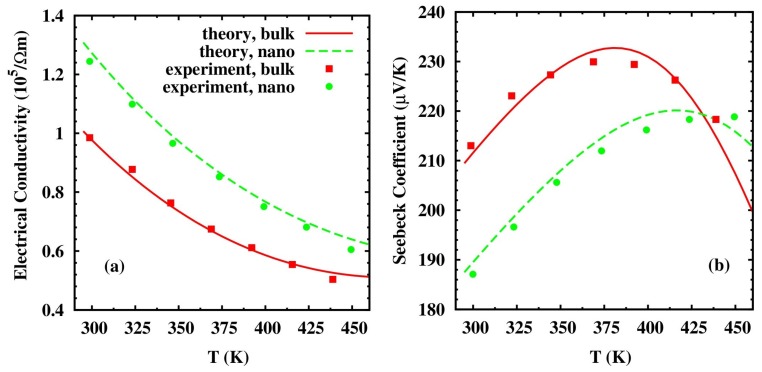
Calculated transport properties for state-of-the-art *p*-type Bi_0.5_Sb_1.5_Te_3_ alloy and the corresponding NC materials with respect to temperature, compared with experimental data in Reference [54]. (**a**) Electrical conductivity. (**b**) Seebeck coefficient. Reprinted with permission from Reference [163]. Copyright 2010, American Physical Society.

Figure 18 shows the comparison of calculation results of Zhou et al.’s transport model with the experimental data of *p*-type Bi_0.5_Sb_1.5_Te_3_ alloy and high efficiency BiSbTe alloy-based NCs: (Bi_0.14_Sb_1.86_Te_3_)-(Bi_0.5_Sb_1.5_Te_3_) NC reported by Poudel et al. in Reference [54]. The calculation results of both bulk alloy material and NC material fit very well with the reported experimental data from 300 K to 450 K. The fitting parameters used in calculation are in good agreement with reported values in handbooks and other references [163]. In Figure 18 (a), (b), the calculated electrical conductivity and Seebeck coefficient agree well with the experimental data by choosing an effective barrier height $E_{b}\approx 0.05$ eV, the average size of particles a = 20 nm as reported in Reference [54], and the volumetric fraction of nanoinclusion *x* = 0.06 as fitting parameters.

The size dependence of electrical conductivity, Seebeck coefficient, and power factor for two different (Bi_0.5_Sb_1.5_Te_3_)_1−*x*_-based NCs with volumetric fractions, *x* = 0.1 and *x* = 0.3 have been calculated by Zhou et al. [163]. For a fixed size a, larger *x* means more interfaces in the NCs, which results in higher Seebeck coefficient and lower electrical conductivity for *x* = 0.3 than that for *x* = 0.1 NCs. For a fixed volumetric fraction *x*, electrical conductivity increases and the Seebeck coefficient decreases with the increase of size a since fewer interfaces are involved. The power factor of NCs with both *x* = 0.1 and *x* = 0.3 does not change dramatically with the size a. Therefore, smaller size and larger volumetric fraction of nanoinclusions lead to stronger filtering effect, i.e., smaller electrical conductivity and larger Seebeck coefficient. The power factor could be slightly enhanced by properly choosing the parameters.

The difference between the particle-particle type and particle-host type NC is studied by Zhou et al. [163] through the relaxation time model. The electrical conductivity and Seebeck coefficient of these two kinds of NC are compared in Reference [163] when the size of nanoinclusion is a = 10 nm. It is obvious that the filter effect of particle-particle type is stronger than the particle-host type under the same condition; in other words, the reduction of electrical and the enhancement of Seebeck coefficient are more significant for the particle-particle type when size and volumetric fraction of nanoinclusions are the same.

### 4.3. Band Structure and Relaxation Time Engineering in Nanocomposites

Besides the low-energy carrier filtering effect we discussed in the previous section, possible band engineering using nanostructures is another promising way to improve the TE efficiency of NCs. According to the theory proposed by Mahan et al. [182], Zhou et al. [183], and Jeong et al. [184], sharp transport distribution functions, which could be induced by band structure with sharp DOS or sharp feature in the relaxation time versus energy [185], could result in an optimal TE figure of merit. Therefore, the modification of band structure and relaxation mechanism in NCs with specific geometries which results in a narrow transport distribution function is preferred.

**Figure 19:**
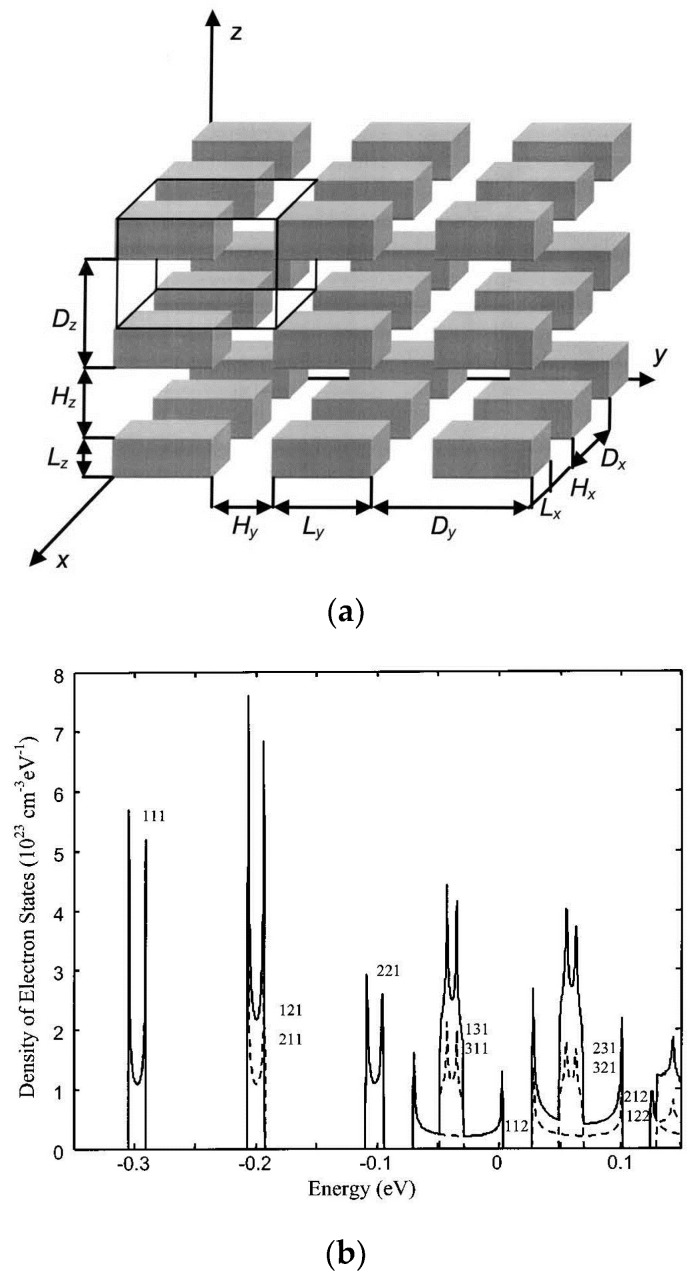
(**a**) Schematic structure of the orthorhombic quantum dot (QD) NCs. (**b**) Density of states of heavy hole of each miniband calculated in Ge/Si QD NCs with the parameters *L_x_ = L_y_ =* 5 nm, *L_z_* = 2.5 nm, and *H_x_ = H_y_ =* 2.5 nm, *H_z_ =* 1.25 nm shown with the dash line. The solid line shows the total DOS. Reprinted with permission from Reference [190]. Copyright 2002, American Institute of Physics.

The promising nanostructure which leads sharp electron DOS is the QD-based “*ordered particle-host-type*” NCs, for example, PbSe QD superlattice fabricated by Wang et al. [186] exhibited very high Seebeck coefficient. Theoretically, Fomin and Kratzer [187] studied the TE transport in periodic one-dimensional stacks of InAs QDs in GaAs matrix using the effective-mass model and BTE. In their model, both the miniband formation and the modification of relaxation time due to quantum confinement effect on the deformation potential acoustic-phonon scattering are considered. Balandin and Lazarenkova [188,189,190], Yadav et al. [191], Khitun et al. [192] modeled the formation of minibands with sharp DOS in NCs with cubic or cuboid QDs periodically embedded in matrix by solving Schrödinger equation under the envelope function effective-mass approximation. BTE with constant relaxation time [182,185] or relaxation time in bulk materials [186] is used to calculate the transport properties. An enhancement of Seebeck coefficient is found due to the miniband formation. Figure 19 shows the orthorhombic QD NCs studied in Reference [190] and the calculated sharp DOS of minibands in Ge/Si QD NCs.

Other methodologies to engineer the band structure or scattering mechanism in NCs that do not require rigorous periodic geometric structures are also proposed. For example, Xu and Li [193] used the deformation potential theory and a degenerate **k·p** method to investigate the strain effect of *n*-type Si/Ge NCs which could induce energy band shift and effective mass variation. Popescu and Woods [194] investigated electronic structure modified by the locally distorted DOS induced by spherical nanoinclusions embedded in bulk PbTe. The mechanism in this model is analogy to the DOS distortion induced by resonant impurity doping [43]. Significant TE efficiency enhancement is found for strongly localized DOS modification at Fermi level.

**Figure 20:**
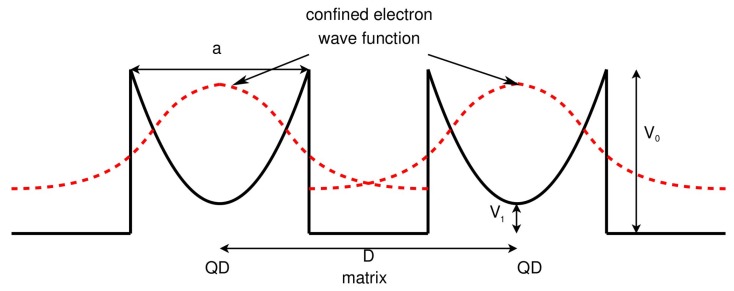
Schematic diagram of the band structure of QDs and the quantum confinement of electron wave functions. *V*_0_ is the height of confinement potential and *V*_1_ is the difference of band edges between QD and matrix. Reprinted with permission from Reference [195]. Copyright 2011, American Institute of Physics.

One particular case of the ordered particle-host type NCs is that the nanoinclusions are QDs with electron confined inside, so-called QD NCs. In this NC material, both band structure and relaxation time engineering could be realized. For finite confinement potential, the electron wave function of the quantum confined electrons would extend into matrix as shown in Figure 20. These electrons would hop across QD arrays. A tight-binding together with Kubo formula and Green’s function method is established by Zhou and Yang [195,196,197]. They wrote the second quantized Hamiltonian of the QDs arrays as [198]:

(15) $$H=\sum_{l,\theta }E_{l}c_{l,\theta }^{†}c_{l,\theta }-\sum_{l,\theta ,\theta^{\prime }}J_{l}\left(R_{\theta^{\prime }}-R_{\theta }\right)c_{l,\theta^{\prime }}^{†}c_{l,\theta }=\sum_{l,k}E_{l}\left(k\right)c_{l,k}^{†}c_{l,k},$$

where $c_{l,\theta }^{†}\left(c_{l,\theta }\right)$ is the creation (annihilation) operator at position θ in miniband *l*, $c_{l,k}^{†}\left(c_{l,k}\right)$ is the creation (annihilation) operator with momentum **k**, $E_{l}$ is the energy of the *l*th level. The overlap integral $J_{l}\left(R_{\theta }-R_{\theta^{\prime }}\right)=J_{l}\left(\delta \right)$ in Equation (15) which describes the hopping strength of electrons between two QDs is $J_{l}\left(\delta \right)=-\int^{\text{​}}dr\phi_{l}^{*}\left(r-\delta \right)\left[V\left(r\right)-v_{0}\left(r\right)\right]\phi_{l}\left(r\right)$ [198] where $V\left(r\right)-v_{0}\left(r\right)$ is the difference between the periodic potential and the potential of individual QD. The electron wave functions confined in QDs are obtained by solving the Schrödinger equation. The transport coefficients could be calculated for a given chemical potential *µ* [199,200]:

(16) $$L_{\mathrm{QD}}^{v}=Ne^{2-v}\int^{\text{​}}\frac{dE}{2\pi }\Xi \left(E\right){\left(E-\mu \right)}^{v}\left[-\frac{\partial f\left(E\right)}{\partial E}\right].$$

Here *e* denotes the charge of carrier, *E* is the energy of electrical carriers, v is integer number, and f(E) is the equilibrium Fermi-Dirac distribution. Factor *N* on the right hand side of the equation denotes the *N*-folded degeneracy of the bands. Then the electrical conductivity, the Seebeck coefficient, and the electronic thermal conductivity in QDs can be obtained as:

(17) $$\sigma_{\mathrm{QD}}=L_{\mathrm{QD}}^{0},$$

(18) $$S_{\mathrm{QD}}=L_{\mathrm{QD}}^{1}/TL_{\mathrm{QD}}^{0},$$

(19) $$\kappa_{c,\mathrm{QD}}=\left[L_{\mathrm{QD}}^{2}-{\left(L_{\mathrm{QD}}^{1}\right)}^{2}/L_{\mathrm{QD}}^{0}\right]/T.$$

The transport distribution function (TDF) Ξ(E) in Equation (16) is:

(20) $$\Xi \left(E\right)=\underset{l}{\sum }\int^{\text{​}}dE^{\prime }{\tilde{\rho }}_{l}\left(E^{\prime }\right){\left|G_{l}^{ret}\left(E,E^{\prime }\right)\right|}^{2},$$

where the square of the module of the retarded Green’s function is ${\left|G_{l}^{ret}\left(E,E^{\prime }\right)\right|}^{2}\approx 2\delta \left(E-E^{\prime }\right)\tau_{l,\mathrm{QD}}\left(E\right)$ [201] under the relaxation time approximation, $\tau_{l,\mathrm{QD}}$ is the total relaxation time of the *l*th miniband in QDs. The quantum confinement effect on the relaxation time has been considered through introducing form factor. ${\tilde{\rho }}_{l}\left(E\right)$ is the effective DOS for transport properties.

**Figure 21:**
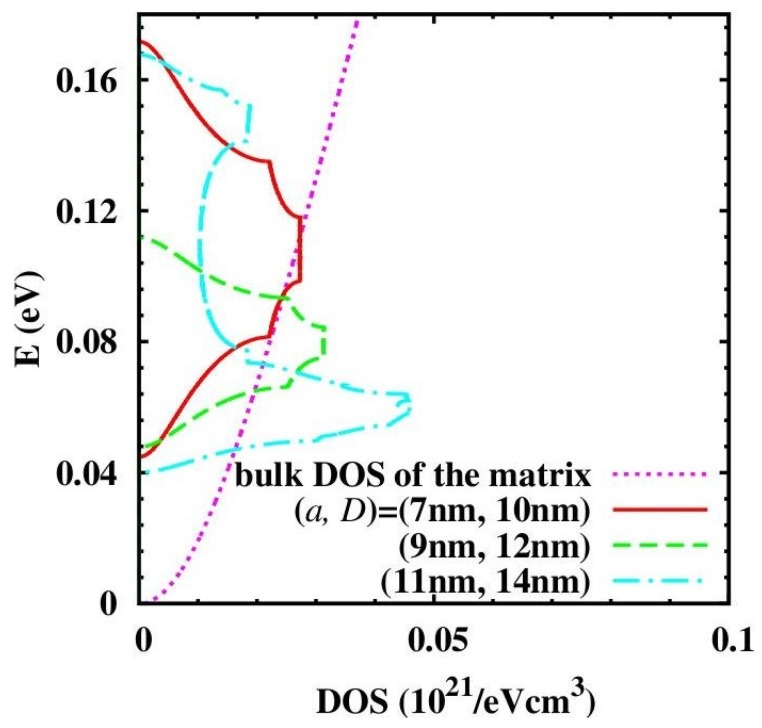
Density of states (DOS) for QDs with varying (a, *D*) in comparison with DOS in the matrix material. Reprinted with permission from Reference [195]. Copyright 2011, American Institute of Physics.

The quantum model is applied to PbSe/PbTe QD NCs. The electron DOS for different QD size a and inter-dot distance *D* in comparison with bulk DOS is shown in Figure 21. The inter-dot spacing *D*−a is fixed to 3 nm. The center and the width of the band are tunable by changing QD size a. When a is small (a = 7 nm and 9 nm), only the lowest miniband appears, since the energy of miniband is inversely proportional to the size of QD. The energy of the higher energy levels could exceed the confinement potential. The center of the miniband shifts from 0.1 eV to 0.08 eV and the band width decreases from 0.12 eV to 0.06 eV when a increases from 7 nm to 9 nm. For larger a (a = 11 nm), higher minibands appear. The DOS becomes sharper when *D* increases since the band width is proportional to the overlap integral, which decays with the inter-dot spacing *D*−a. Such tunability of the center and width of the miniband shown in Figure 21 render possibilities to manipulate the Seebeck coefficient and thus the *ZT* of QD NCs.

The phonon bottleneck effect is found to be possible to engineer relaxation time. By comparison with the relaxation time of the corresponding bulk materials in Reference [195], the total relaxation time in QD NC is a little smaller than that of the bulk material near the lower edge of the miniband and much larger than the bulk case near the upper edge of the miniband for QD NC when (a, *D*) = (7 nm, 10 nm) and (9 nm, 12 nm), respectively. It should be noted that there is no physical meaning for relaxation time out of the edge of the miniband. Although the electron relaxation time near the lower edge is reduced by an order of magnitude, the average relaxation time is indeed enhanced because the increase of the relaxation time near the upper edge is two orders of magnitude. For (a, *D*) = (7 nm, 13 nm), the electron relaxation time of all the energies within miniband is much larger than that in the bulk material. The increase of the electron relaxation time is a result of the restriction of energy and momentum conservation in electron-phonon scattering, i.e., phonon-bottleneck effect for quantum-confined electrons [202]. The effective relaxation time of electron-phonon scattering increases due to the quantum confinement of electrons in QDs.

Zhou et al. [195] combined the classical transport of bulk-like electrical carriers (Section 4.1 and Section 4.2) and quantum hopping transport of quantum-confined electrons described in this section as a two-channel transport model in QD NCs. In this model, the low-energy carrier filtering effect, band structure engineering through miniband formation, and relaxation time engineering through phonon-bottleneck effect are considered. The temperature dependence of electrical conductivity and Seebeck coefficient are calculated using two-channel transport model in PbSe/PbTe QD NC for QDs with different size a and inter-dot distance *D*. The electrical conductivity and Seebeck coefficient can be enhanced simultaneously for QD NC with (a, *D*) = (7 nm, 10 nm) and (a, *D*) = (8 nm, 11 nm). The enhancement of electrical conductivity is a result of the large overlap integral of small inter-dot spacing and the increase of relaxation time of electron-phonon scattering due to the phonon-bottleneck effect. For QD NC with (a, *D*) = (9 nm, 12 nm), the electrical conductivity is reduced. The Seebeck coefficient is enhanced at low temperature while reduced at high temperature. In all of the above three cases, the transport channel of quantum-confined electrons dominates. In contrast, the contribution from quantum-confined electrons could be very small for QD NC with (a, *D*) = (7 nm, 12 nm) as the overlap integral dramatically decreases with *D*−a. The electrical conductivity is thus reduced a lot and the Seebeck coefficient is only slightly enhanced due to the classical interface filtering effect.

**Figure 22:**
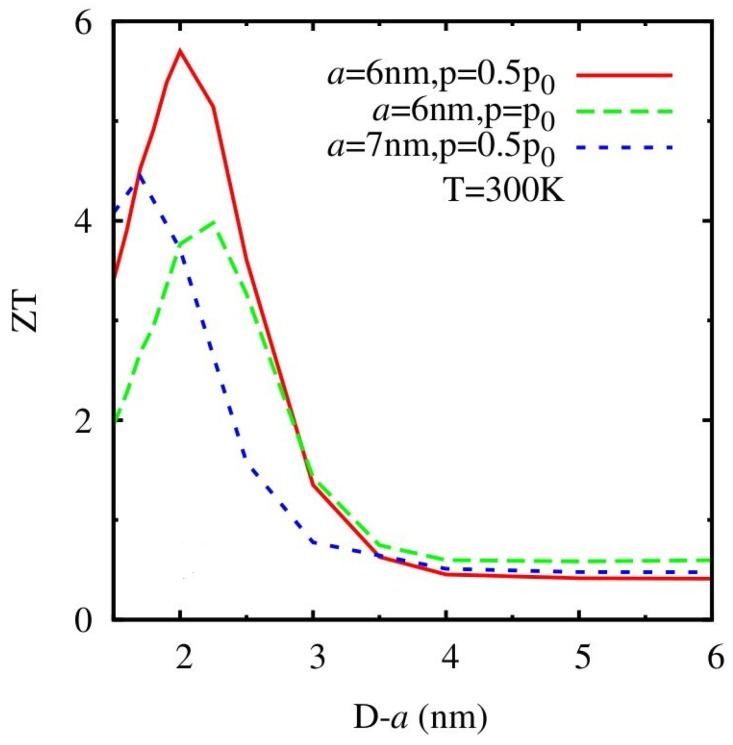
Inter-dot spacing (*D*−a) dependence of *ZT* for QD NCs with different size of QD and different doping concentration at *T* = 300 K. Reprinted with permission from Reference [196]. Copyright 2012, American Physical Society.

The authors further applied the two-channel transport model to find the optimal band width for TE materials pointed out in Reference [183] in QD NCs. Figure 22 shows the dependence of *ZT* on the inter-dot spacing, which is inversely proportional to the band width of minibands, for different QD size and doping concentrations in Bi_2_Te_3_/Sb_2_Te_3_ QD NCs at *T* = 300 K [196]. The maxima of *ZT* are found: (1) when *D*−a is 2 nm for a = 6 nm and *p* = 0.5*p_0_*; (2) when *D*−a is 2.3 nm for a = 6 nm and *p* = *p*_0_; (3) when *D*−a is 1.7 nm for a = 7 nm and *p* = 0.5*p_0_*.

## 5. Summary

In this article, we review the progress in the field of nanocomposite thermoelectric materials, which are nanostructured materials with nanoconstituents embedded in a matrix material or a mixture of different nanoconstituents. It has been theoretically proposed and experimentally synthesized to be a high efficiency thermoelectric material by reducing the lattice thermal conductivity due to phonon-interface scattering and enhancing the electronic performance due to modification of electron scattering and band structures. Nanocomposites materials based on various classes of materials and kinds of models are introduced. It is possible to slightly increase the power factor through low-energy carrier filtering and reduce the lattice thermal conductivity through phonon interface scattering in nanocomposites. In periodic quantum dot nanocomposites, band structure engineering due to miniband formation and relaxation time engineering through phonon bottleneck effect could even lead to a simultaneous enhancement of electrical conductivity and Seebeck coefficient with proper size of quantum dots and inter-dot distance. Optimal band width for thermoelectric properties can be realized in quantum dot nanocomposites.
